# Colonisation of Lunatics by the Legislature

**Published:** 1862-07

**Authors:** 


### Art. III.—COLONISATION OF LUNATICS BY THE LEGISLATURE.

We conceive that the announcement contained in the title of this paper is of great significance and importance; and, in whatever way it may be interpreted and whatever may be the issue, marks a vast advance in public and legislative opinion as to the disposal of certain classes of the insane. But before entering upon the subject, it will be interesting, and may serve to carry forward the materials for such a history of the development contained in the proposed enactment to which we are about to refer, as a Journal devoted to Medical Psychology should contain, to record some further contributions to the mass of information accumulated as to the machinery and movement of the Institution of Gheel.

I.—Brief allusions were made to the subject in a recent discussion in the Parisian Medico-Psychological Society upon the asylums in Holland, when a fitting tribute was paid to the philanthropy as well as to the scientific discoveries of Schrœder Van der Kolk; but in an article, occupying nearly fifty pages, of the April number of the *Annales d'Hygiène Publique,* M. Brierre de Boismont has given a very copious exposition of the bibliographical and practical history of colonisation as applied to the treatment of the insane.* It is, and perhaps was intended to be, supplementary to the report of M. J. Falret, which treated very sparingly of the observations and opinions of former writers. M. de Boismont sees in the treatment of the insane in France two great epochs: that of Pinel, who introduced the principles of humanity and justice as a basis; and that of Esquirol and Ferrus, who advocated the erection of suitable asylums, in which the resources of art, as well as the promptings of philanthropy, could be brought to bear upon those deprived of mental health. But even these developments did not satisfy the cravings of modern sympathy; and Conolly initiated the abolition of all restraint, and Parigot sought in the fresh, free air and family circle means of cure and amelioration. Either the increase in the number of victims, or the more perfect appreciation of the benefits of the hospital, has crowded the asylums provided; and colonisation has been looked to as a remedy and a means of diminishing the expense entailed upon the community. To determine the claims of this expedient is the main object of M. de Boismont, as it was of the commission appointed by the Parisian Medico-Psychological Society upon whose report his more varied observations may be accepted as an illustrative commentary. His memoir is preceded by the usual frontispiece of Gheel, which presents few additional features to those supplied in the descriptions of Duval, Coxe, and his own in 1846; and being taken from Bulckens is naturally more favourable in aspect than the picture depicted by Dr. Macintosh, the last painter of the scene.† M. de Boismont's interesting description is gracefully followed by the reiteration of encomiums upon the population *d'infirmiers-nès,* who, in place of degenerating from continued contact with the insane, have been strengthened in moral dispositions and benevolence. There are given glimpses of various approximations to a

* An epitome of this paper has been given in the *Union Médicale,* and the *Ann. Medico-Psychol.*, Avril, 1862.

† *Asylum Journal,* vol. viii. p. 1—36.

system of colonisation in this country and others, either projected or commenced upon a very humble and tentative scale; in some cases practical, and in others altogether Utopian; and of similar experiments attempted in remote localities in France under private auspices, where the inmates were not exclusively insane, and were committed to the charge of religious bodies. It is added that M. Parigot, *le propagateur de l'idée*, is now carrying out his views in the State of New York; but with conceptions modified, perhaps, by distance, but differing widely from those entertained by him when proposing that asylums should bear upon their portal, *Lasciate ogni speranza voi ch'entrate*, as will be subsequently seen.

In 1842, Guislain regarded Gheel as possessing the following advantages:—1. The freedom in the open air enjoyed by the greater number of the patients. 2. The facilities for occupation and the kind of work afforded to the robust. 3. The moderate average rate of board. 4. The society, the attention, and the kindness of the guardians. But in his estimation these must be contrasted with, as they were assuredly modified by, the following considerations:—1. The necessity of limiting the colony to the reception of incurables and to certain classes even of these. 2. The heterogeneous character of the superintendence. 3. The want of unity in the medical service, and the absence of an infirmary. 4. The low minimum rate of board. 5. The defective means of transport. 6. The indiscriminate distribution of newly arrived patients. 7. The absence of the proper classification even necessary for incurables, and of the separation of the sexes. 8. The disposal of the sick, the ignorance of the French language, the access to stimulants, &c. Even in 1856, when certain of these strictures, which involved grave condemnation, were no longer applicable, M. Guislain still demanded an infirmary; the addition of which it is obvious would in a great measure transform Gheel into an asylum. Two opinions as to the mode in which colonisation could be carried out are contrasted. 1. Either the lunatics could be placed in ordinary and independent cottages, where they would be superintended by the parochial or local surgeons. 2. Or, they could be placed in special cottages under the care of the medical officers of the nearest asylum. While the second may be disturbed by the antagonistic interests of the different officials employed, it is not open to the grave and insurmountable objection that it affords no suitable accommodation nor protection for individuals manifesting dangerous or destructive propensities, no means of treating cases of acute disease. While Falret sees in colonisation properly administered, a solution of the problem as to disposing of the accumulation of chronic lunatics; he has a greater difficulty in discussing the questions, can it be extended to all classes of the insane, and can it be transplanted into the soil of France? After consideration that the special aptitude and training and religious tendencies of the Gheelois cannot be secured, nor a substitute obtained; that the elements of the French character are little adapted for such a mission; and that, though unmitigated claustration is convenient for the guardians, but evil for the insane, yet without an asylum there exists no provision for suicides, homicides, incendiaries, refusers of food, nudifiers, elopers, &c.; he comes to the conclusion that an asylum is indispensable, as a part and centre of any plan of colonisation that can be introduced among his countrymen.

To reinforce more obvious and practical arguments, M. de Boismont magnifies what he conceives the triumphs of moral medicine. He represents Guislain as winning from profound melancholy, mutism, and lethargy a girl, by his gentleness, his efforts to rouse, encourage, and inspire with hope; and Leblond as dissipating the delusion of a maniacal old soldier, who imagined himself Napoleon, and who complained of the insolence of the attendants towards their emperor, by the remark, "Yes, you are Napoleon, but Napoleon at St. Helena." These are not felicitous illustrations of the indispensability of a special arrangement, or mission, in managing the insane. They do not involve the necessity of seclusion of any kind, nor the instrumentality of a physician; but tend rather to countenance that recent heresy which confers an undue prominence and importance upon moral agents, which obscures, if it does not exclude, the grand truth which lies at the bottom of all treatment of the insane, that insanity is a symptom of a disease of structure; and which suggests the notion that if affections of the nervous system are amenable, exclusively or chiefly, to amusement, education, appeals to reason, or religion, or the sense of the ludicrous, such means of cure or alleviation could be better, more dexterously, and delicately applied by men not necessarily belonging to the medical profession, but who have made the human mind a matter of special investigation.

In the inquiry as to the different classes of lunatics who could be safely and advantageously placed in a colony, of which the medical officer at the head of the asylum would be the mainspring, it is gratifying to remark a great similarity to the remarks and conclusions which have recently appeared in this Journal. M. de Boismont conceives that convalescents, tranquil maniacs, monomaniacs, and melancholics without evil tendencies, dements, inoffensive epileptics, and certain imbeciles and idiots might be entrusted to the cottagers; and recognises in such a provision an extension of the pecuniary aid given to discharged lunatics in Paris. He even hopes that such an arrangement may lessen the public burdens, in so far as the labour of the colonists may contribute to their own maintenance; and argues that it is but fair to call upon even the lunatic to pay his quota into the general treasury. He conceives that a community of a thousand individuals would be required to give efficacy to such a plan. He further holds that it would be impossible to enclose such an establishment from the enormous outlay required; but that, wherever it be placed, it must be morally dependent and connected with the asylum; and enters, at considerable length, upon the economy and success of the colony of Fitz-James at Clermont, Oise, which must now be well known to English readers. So conspicuous is field labour as a part of the system there pursued, and doubtless as a source of the pecuniary gain, that he feels it incumbent upon him to rebut supposed objections to white slavery. He, however, forgets that the real objection to servile labour in private speculations for the lodgment of the insane, is not to occupation as a means of cure, but to the kind selected, the amount imposed, and the inordinate profits which accrue. M. de Boismont concludes in the following terms:—"Mais si nous donnons notre approbation au systême de la colonie, nous nous empressons de reconnoître que les individus mineurs ne peuvent jouir de même droits que les citoyens libres; ainsi, et c'est notre conclusion, n'admettons-nous actuellement le colonie qu'avec le voisinage de l'asile et la surveillance du Médecin Directeur."

II.—In a letter, dated 10th March, M. Parigot, "le propagateur de l'idée," writes from his adopted home at Sing-sing, New York:

"United States, N.Y., Sing-sing, 10th March, 1862.

"My dear sir, and much respected friend—Your very kind letter of the 1st of February has been duly received; I remember also with much pleasure the too short time spent in your company, and do keep a vivid memory of your kindness to me. As you perceive, I left Belgium with my family, never to return to live in that country.

"I see with much pleasure that new asylums are under erection in Scotland, on the principles you advocated in 1860. I will be happy to hear of success in establishing this new form of asylums, approaching the system of real colonies; but allow me to say I do not think you or your friends will derive any profit from your visit to Gheel, whose great principle of devotion to humanity is fast declining in spite of the good-will and the assertions of Dr. Bulckens in his last report. Anciently, as you know, great abuses existed in that colony; the peasant was *plundered* by the then existing brokers in lunatics; the form was generally bad, but, *individually*, charity was often a real fact; then, also, some cities and towns had honest supervisors in Gheel, who, by their dealings and doings, poured comparatively shame on the bad ones; it was a source of emulation and even of forced competition. When, in 1849, I was first appointed there, it required a long time before I could appreciate the real value and its relative *effet utile* of such a colony. Its mechanism was simple, and to describe it I found no better expression than *air libre et vie de famille*. From that time, my only aim was to develope and point out the morality and charity of such institution, and to make it the general principle of Gheel; and now, my best reward is to know and to be conscious, that at least in that point I have been useful. In the plans explained in my little pamphlet on Gheel, an infirmary was to be built at a certain distance off the village (above a mile or more), far from cafés and beerhouses, and that building was to be a simple reproduction, on a large and convenient scale, of a family mansion, being the centre of medical action. Besides all things necessary for such purpose, I wanted large grounds, somewhat elevated, where I could have pure, dry air, and almost pure distilled water, from rivulets originating at the foot of sandy hills in the neighbourhood, and that water in sufficient quantity for the bathing of 800 to 1000 patients! Large fields should have belonged to the infirmary, and everything was to be so arranged as to permit an imperceptible transition from a large farm to an hospital.

"Now, my dear sir, under the inspiration of the late Guislain, who had quite different opinions on the utility of a colony, and was an open and honest enemy to Gheel, but the consequence of which was a mistake, and actually under the influence of some hypocrites, or *faux bons hommes* as they are called, who knew very well that the surest means to destroy Gheel is to alter its characteristics, they have built the *infirmerie*, you will have to examine, with its *cells Guislain*, *en forme de menagerie*, &c. &c., and established a *comité* which has the whole moral and material management of the colony. Inquire, if you please, of some Gheelois who are these men, their profession, avocations, &c., and perhaps you will find that they are the very land and house proprietors, notaries, shopkeepers, &c., who in reality divide amongst themselves the benefit of keeping the insane. There may be an attempt to make you believe that the physicians have the moral direction of the institute, but perhaps some will say the truth.

"Now, I can say that the *secret* of the future *Gheels* as colonies, or as *open asylums*, as I would call your modification in Scotland, depends entirely and from no other condition than the spirit of *justice and morality*, and *devotion* to humanity, of their superintendents. These fundamental principles should impregnate the atmosphere of the asylums, farms, or cottages. The next great necessity, as you are well aware, is the choice of proper individuals as *attendants*—*nouriciers*, &c., as you may call them.

"I need not to mention the *great art* and *difficulty* of adjusting the various qualities and even *defects* of the sane and insane persons who are to live together. All these considerations lead me to say, that, if I had the charge here or anywhere else to establish such colonies, I should, first of all, instruct a small number of attendants (saturate the air with good principles), who would instruct and show the example to others, and then afterwards gradually admit the patients. It is a very difficult period to pass, but once the machine is set to go on good principles—its *effets utiles* being perceptible to all—every inmate, in his capacity, will help the regular action of the institution.

"To resume. I believe that Gheel, changing its characteristics, is still the most imperfect of asylums, especially when compared to your beautiful asylums. It declines as a colony, because it loses its original simplicity and in some degree devotion to the insane. Now it is in the hands of *doctrinaires*, without principles but that of filling their pockets. It is true they have the numerous *reglements*, but they will never do until the colony is not an asylum.

"J. PARIGOT."

"P.S.—Our political sky becomes clearer for the present, but I fear the heaping up of difficulties between this country and Europe for the future."

We regard these statements as most valuable. They are the views of an enthusiastic admirer and advocate of Gheel, looking at the scene of his labours and success from the outside; they are the utterances of a partisan who has become familiar with other manifestations of benevolence, other modes of attaining the objects which he had at heart, and since he adopted his creed; they are the legacy of one dead to European civilization, and surrounded by new social arrangements, by a moral atmosphere of heavy pressure, rapid circulation, and with an intense tendency to foster new and extravagant forms of organization.

III.—In a Bill now in Committee in Parliament, and ordered to be printed 12th May, designated a "Bill to make further provision respecting Lunacy in Scotland," there is to be found the following clause:—

"5. It shall be lawful for the Board to grant special licences to occupiers of houses, for the reception and detention therein of lunatics, not exceeding four in number, subject to such rules and regulations as the Board may appoint, and to exempt the holders of such special licences from the payment of any fee, or of any sum whatever in respect thereof; and except in so far as expressly exempted by the Board, the holders of such licences shall be subject to the whole provisions applicable to the keepers or superintendents of private asylums in the recited Acts and this Act contained; and the Board, in the case of a lunatic who is a pauper, on the application of the Inspector of Poor of the Parish liable at the time for the maintenance of such lunatic, or in any other case, on the application of any one legally entitled to make the same, accompanied by medical certificates in the forms hereinafter prescribed, may sanction the reception and detention of such lunatic in any house so specially licensed; Provided that no lunatic shall be received into any such house without the sanction of the Board, granted according to the forms and regulations approved of by them; and any person receiving any lunatic into any house specially licensed as aforesaid, or being concerned in the disposal of such lunatic without the sanction of the Board, shall be liable to a penalty not exceeding ten pounds."

We think that these lines may be traced either to the direct instrumentality of the medical members of the Board of Lunacy, Scotland, or to the influence of their writings, and above all of the opinions advocated in the annual reports of the body to which they belong, exercised over the legal and representative element in their community. Be this as it may, here is a proposal to withdraw lunatics from the private houses and the irresponsible superintendence to which they are at present consigned, and to place them in small licensed asylums subject to the regulation and visitation of public officers. Such an arrangement has even wider relations than what the framers of the clause contemplated. It will become not merely a measure for placing certain classes of the insane under favourable circumstances for supervision; but it may be rendered subservient to the establishment of suitable accommodation and treatment succursal to the district or central asylum. There exists every probability that as no licence-fee is to be exacted, and as the curators will accordingly be enabled to receive inmates at a lower charge than the proprietors of private, and from the economic scale of the establishment, than the authorities of district or public asylums; pecuniary reasons may induce Parochial Boards to prefer such a residence to every other for pauper lunatics, whatever the form or stage of their malady may be. The indiscreet exercise of such a disposition will, however, be counteracted by the powers vested in the Board of Lunacy to determine, first, whether an individual requires asylum or cottage treatment; secondly, the nature and amount of accommodation; and thirdly, the suitableness of each case for the particular house and family. But even should patients be intrusted to these depôts whose condition requires curative efforts, or something more than the perfunctory quarterly visits prescribed to the parochial medical officers, there exists in the Act now amended a provision by which District Medical Inspectors may be appointed, whose duties either comprehend, or could easily be made to comprehend, the physical and moral treatment of the unconfined insane.* But it may be fairly anticipated that into such cottage asylums will be gathered not merely the outlying, and deserted, and friendless, as well as the chronic and incurable insane from the adjoining hills and hamlets, but that they will receive patients recently discharged from asylums, either during that probationary period most wisely made legal and practicable by the 13th clause of Bill 120, 25 Vict.; or as a new and untried mode of amelioration; or because they have no relatives nor friends to whom they can be intrusted, when the disease is found to be intractable.

Such cases would likewise naturally fall to the charge of the

* Cl. LXX. 20 & 21 Vict. cap. 70.

District Inspectors. But should the inertness or the parsimony of the ratepayers defeat this project, it appears to be competent to the Board, in carrying out such a principle, to render the licensed houses directly connected with and subordinate to asylum management and influence. The site or selection of the house is not restricted to the parish to which the pauper lunatic belongs; in fact, such a limitation would be impracticable, as many parishes have only one pauper chargeable, and many more only one in such a situation as to admit of such an allocation; so that these sodalities must be formed by the union of several parishes; may be placed according to sanitary or other considerations; and may, finally, be grouped at convenient distances around the parent or presiding asylum; and subjected to as much or as little of its influence, and to the government, or merely the supervision of the officials, as appear expedient. It does not appear upon whose application the special licence required for such houses is to be granted; but as the approbation and sanction of the Board of Lunacy is to be given, according to certain rules and regulations, the precise point from which the initiative is taken is of little moment. Whether it be from the Parochial Board, or from the individual proposing to be the guardian, due inquiries will be made, the necessary guarantees will be demanded from the clergymen, the heritors, or inhabitants of the locality, as to the moral character and general fitness of the applicant for the trust which it is proposed should be confided to him. He cannot be much inferior to the retired or bankrupt butcher or baker who, it is affirmed, formerly usurped this office in Scotland; and we hold that he must be infinitely superior to the custodiers who at present obtain the charge of the unconfined insane in that country, at haphazard, and rather in consideration of the size or solitariness of their cottage, than of their personal qualifications.

It might be rash to affirm that the persons at present so employed do not belong to the best class of the peasantry, that their poverty and not their will consents to the painful and disagreeable duties which they undertake; but it may be safely advanced, and without derogating from their respectability, that moral qualifications never enter into the contract which they have formed. The certificates or guarantees which we have supposed will be exacted as conditions entitling to such a licence should refer to the manner in which the applicant has fulfilled the social and domestic relations of life as much as to intelligence, or sobriety, or industry, because home and the home circle are the soil and sphere in which gentleness, and humanity, and forbearance most generally flourish, and are the best tests of the power and permanence of such sentiments. Such candidates will, at all events, be free from all previous prejudices and errors; and in so far as they may thus be found willing and prepared to act submissively and earnestly in accordance with such instructions as may be furnished, they will be found exempt from the self-will and hereditary opinionativeness of the *gardiens-nès*. For, even if the boasted aptitude to treat the insane of the cottagers of Gheel be admitted, it is impossible to avoid the corollary of such admission, that they can inherit nothing more nor less, nor better than what their ancestors possessed and had to transmit. They are the immediate representatives, the pupils, the heirs of a system characterized by some cruelty, considerable neglect and coercion, and much superstition. They stand forth, according to certain of their advocates, gentle, pure, disinterested, from a harsh, and dark, and corrupt background. They are passion-flowers springing from a dunghill. And yet, if Dr. Macintosh be correct, certain of the ancestral usages have been perpetuated.*

There may be disadvantage in the faulty internal construction of the cottages, which may be offered to the consideration of the Board; but there is an avoidance of delay and many minor obstacles in taking them as they are; and from the number of competing domiciles which it may reasonably be expected will be at the disposal of the Board for selection, those of superior accommodation may be preferred; while the situation and surroundings may, to a certain extent, be pastoral or arable, inland, or mountainous, or littoral, according as the existing condition or previous habits of the lunatic may make advisable.

The phraseology of the second paragaph of the clause quoted, would imply that power is to be vested in the Commissioners to exempt the holders of such houses from certain restrictions to which the keepers of private asylums are subject; but it may be prudent to incorporate in the rules and regulations to be prepared, directions as to the nature and duration of the occupation in which it may be permissible to engage the inmates. It was Sir Andrew Halliday, we think, who claimed for Scotland a priority in introducing physical labour as a mode of treatment in insanity. A farmer, actuated perhaps by a selfish desire to economize paid labour, cultivated his fields by the assistance of lunatics boarded in his house, and discovered that in reclaiming waste land they cured themselves. The ruralization of retreats for active and robust members of this class appears a fitting occasion for extending the application of this discovery; but while we trust that the scheme may spread as rapidly and successfully as it has done in claustral asylums, it may be necessary to protect the workman from a tax upon his strength and endurance, which the active and robust sane alone can pay and pay with difficulty, but

---

* *The Journal of Mental Science*, April, 1862, p. 20.

which the appendices of the Scotch Board afford abundant evidence is now imposed and exacted in lieu of more liberal parochial support. Such a precaution may appear still more necessary should this arrangement be made to include—as from the construction of the fifteenth and sixteenth lines we conceive it may include—the independent and self-maintaining lunatic. In so far, however, as these individuals will not be removed beyond the cognizance and authority and visitation of constituted public officers upon the one hand, and, should a connexion with the district asylum be established, from the management of trustworthy experts upon the other, the very process by which the plethora of the overgrown asylum is relieved will not be by the sudden and random or experimental withdrawal of aged and effete and incurable cases, but may be regulated by a consideration of the benefit and capacities of each individual.

---

